# Methane-Carbon Flow into the Benthic Food Web at Cold Seeps – A Case Study from the Costa Rica Subduction Zone

**DOI:** 10.1371/journal.pone.0074894

**Published:** 2013-10-07

**Authors:** Helge Niemann, Peter Linke, Katrin Knittel, Enrique MacPherson, Antje Boetius, Warner Brückmann, Gaute Larvik, Klaus Wallmann, Ulrike Schacht, Enoma Omoregie, David Hilton, Kevin Brown, Gregor Rehder

**Affiliations:** 1 Department of Environmental Sciences, University of Basel, Basel, Switzerland; 2 Max Planck Institute for Marine Microbiology, Bremen, Germany; 3 Sonderforschungsbereich 574, University of Kiel, Kiel, Germany; 4 Helmholtz Centre for Ocean Research Kiel, GEOMAR, Kiel, Germany; 5 Centro de Estudios Avanzados de Blanes (CEAB-CSIC), Blanes, Spain; 6 Alfred Wegener Institute for Marine and Polar Research, Bremerhaven, Germany; 7 Centro de Astrobiología (CSIC/INTA), Instituto Nacional de Técnica Aeroespacial Torrejón de Ardoz, Madrid, Spain; 8 Scripps Institution of Oceanography, University of California, San Diego, United States of America; 9 Leibniz Institute for Baltic Sea Research Warnemünde (IOW), Rostock, Germany; Wageningen University, The Netherlands

## Abstract

Cold seep ecosystems can support enormous biomasses of free-living and symbiotic chemoautotrophic organisms that get their energy from the oxidation of methane or sulfide. Most of this biomass derives from animals that are associated with bacterial symbionts, which are able to metabolize the chemical resources provided by the seeping fluids. Often these systems also harbor dense accumulations of non-symbiotic megafauna, which can be relevant in exporting chemosynthetically fixed carbon from seeps to the surrounding deep sea. Here we investigated the carbon sources of lithodid crabs (*Paralomis* sp.) feeding on thiotrophic bacterial mats at an active mud volcano at the Costa Rica subduction zone. To evaluate the dietary carbon source of the crabs, we compared the microbial community in stomach contents with surface sediments covered by microbial mats. The stomach content analyses revealed a dominance of epsilonproteobacterial 16S rRNA gene sequences related to the free-living and epibiotic sulfur oxidiser *Sulfurovum* sp. We also found *Sulfurovum* sp. as well as members of the genera *Arcobacter* and *Sulfurimonas* in mat-covered surface sediments where *Epsilonproteobacteria* were highly abundant constituting 10% of total cells. Furthermore, we detected substantial amounts of bacterial fatty acids such as i-C15∶0 and C17∶1ω6c with stable carbon isotope compositions as low as −53‰ in the stomach and muscle tissue. These results indicate that the white microbial mats at Mound 12 are comprised of *Epsilonproteobacteria* and that microbial mat-derived carbon provides an important contribution to the crab's nutrition. In addition, our lipid analyses also suggest that the crabs feed on other ^13^C-depleted organic matter sources, possibly symbiotic megafauna as well as on photosynthetic carbon sources such as sedimentary detritus.

## Introduction

Most deep-sea ecosystems on Earth are considered to be energy limited, because they depend on a small fraction of photosynthetically produced organic carbon (C), which sinks from the productive ocean surface to the seafloor [1], [2]. They are contrasted by chemosynthetic ecosystems such as hydrothermal vents and seeps, which are fueled by chemical energy transported with subsurface fluids. Especially cold seeps, which form around mud, gas and oil escape structures and which are characterized by high methane effluxes [3], [4], support high biomasses of deep-sea life, comprising chemosynthetic microbial mats and megafauna as well as associated heterotrophic animals [5]–[9]. The key biogeochemical process at cold seeps is the anaerobic oxidation of methane with sulfate (AOM), which is a net conversion of methane and sulfate to carbon dioxide and sulfide [10]–[12]. The seeping sulfide fuels aerobic thiotrophic communities comprising free-living and symbiotic bacteria. The free-living thiotrophs often form dense microbial mats above gassy sediments [13]–[15]. Symbiotic megafauna such as bathymodiolin bivalves and siboglinid tubeworms host thiotrophic bacteria in specialized cells and tissues [7]. Oxidised cold seep surface sediments may also support free-living aerobic methanotrophs [16]–[19]. These are not known to form dense mats at cold seeps, but they also occur as endosymbiotic associations with megafauna, such as bivalves and tubeworms [20]–[23]. Furthermore, some highly adapted, hydrothermal vent or cold seep endemic annelids [24], gastropods [25] and crustaceans [26], [27] farm chemosynthetic, microbial epibionts on their skin and shells, which they graze upon.

An important question in the ecology of vent and seep ecosystems remains as to how chemosynthetically fixed carbon is transferred to the deep-sea food web [5], [9], [28]–[30]. Current knowledge is mostly based on measurements of the stable C isotope ratio of faunal bulk tissue [5], [9], [31], [32]. At cold seeps, both methane and its oxidation product CO_2_, are strongly depleted in ^13^C [33]. Consequently, methanotrophic and thiotrophic bacteria, which incorporate ^13^C-depleted methane and/or ^13^CO_2_ in their biomass are characterized by δ^13^C-values much lower than −15 to −30‰, which is the range typical for photosynthetically fixed C [33], [34]. Consumer species feeding on free living chemosynthetic bacteria or symbiotic fauna hosting these microbes in their tissue will also incorporate the ^13^C-depleted C in their biomass [5], [35]. A valuable addition to the measurement of bulk tissue is the analysis of compound-specific δ^13^C-values, for example of fatty acids (FAs), which are contained in cellular membranes [36], [37]. These lipids are incorporated from the food sometimes without significant alteration into the consumer biomass; e.g. essential fatty acids [38]. Furthermore, some lipids are diagnostic biomarkers because they are synthesized by specific source organisms. The analysis of their presence and specific C-isotope composition help to identify multiple dietary C-sources utilized by a consumer. However, the isotopic composition of biomass typically integrates over significant parts of an organism's lifetime. In order to investigate food sources that a consumer ingested only recently, the analyses of stomach content, including DNA, are frequently used in food web studies [39]–[41].

The aim of this study was to assess the importance of CH_4_-derived carbon for a dominant consumer – lithodid crabs – of the benthic food web at an active cold seep of the Costa Rica subduction zone. We combined bulk- and compound-specific stable C isotope analyses of muscle tissue and stomach contents as well as fluorescence in situ hybridization and screening for microbial 16S rRNA gene sequences to investigate the relevance of chemosynthetically-derived carbon for the crab's nutrition.

## Materials and Methods

### Site description

Mound 12 (Md. 12) is an active mud volcano located at the Central America convergent margin off the coast of Costa Rica at 1020 m water depth (8° 55.85′ N, 84° 18.75′ W; [42]). It belongs to a series of cold seeps along the Costa Rican Pacific margin, which are related to the subduction of the Cocos plate and erosion of continental material, subsequent dehydration of subducted clay minerals as well as production of thermogenic CH_4_ [43]–[45]. At Md. 12, CH_4_, geofluids and mud ascend to the seafloor along faults, which cut deeply through the basement and upper plate sediments [46]. Diapirism and mudflows have formed a roundish (∼800 m diameter) cone-shaped relief (<30 m) with an irregular pinnacle in the NE and a lower profile ridge in the SW [42], [47]. The mudflows are intercalated with slope sediments, indicating that Md. 12 is frequently active, alternated by low-activity phases. At present, the mound seems to be most active at its pinnacle and the SW flank, which is characterized by dense microbial mats and other chemosynthetic organisms (mytilid mussels and Lamellibrachia tube worms) [47]–[49]. At a microbial mat site, we previously measured a total CH_4_ flux of ∼ 10 mol m^−2^ yr^−1^ of which only half was oxidized with SO_4_^2−^ [47]. Indeed, bottom waters above Md. 12 were enriched in CH_4_ with 1–2 orders of magnitude higher concentrations compared to background values, indicating that a significant fraction of the seeping CH_4_ can escape into the water column [49].

### Sea floor observations and sampling

We visited Md. 12 during two consecutive cruises with R/V Atlantis (AT11-28) and R/V Meteor (M66-2) in June and September 2005, respectively. Direct and/or video observations were carried out in June with DSV Alvin (Woods Hole Oceanographic Institute, USA) and in September with ROV Quest (Marum, Germany). In addition, we also photographed the sea floor during cruise M66-2 over a time period of 408 hours with a frequency of 2 pictures per hour. For this approach, a downward-facing digital still camera (Ocean Imaging Systems, North Falmouth, USA, 6.1 Mpix) was mounted on a lander frame (Deep-sea Observation System – DOS [48]) resulting in a field of vision of 0.4 m^2^. The lander was deployed on top of a microbial mat (8° 55.69′ N, 84° 18.78′ W), which covered ∼60% of the cameras field of vision.

A specimen of the abundantly observed lithodid crab (see results and discussion section for a taxonomic assessment), which was apparently feeding on microbial mats, was sampled using DSV Alvin's manipulators (8° 55.72′ N, 84° 18.83′ W). The crab was stored in a basket until surfacing of the submersible and directly thereafter photographed and dissected. A tissue sample from a leg muscle and the stomach were removed and frozen at −20°C until further analyses in the home laboratory. A ∼6 m wide sediment strip (8° 55.69′ N, 84° 18.82′ W) covered by the whitish, thiotrophic microbial mats as well as bare sediments 1–2 m adjacent to the microbial mat were sampled by push coring with ROV Quest.

### Taxonomic identification of lithodid crabs

**Figure 1:**
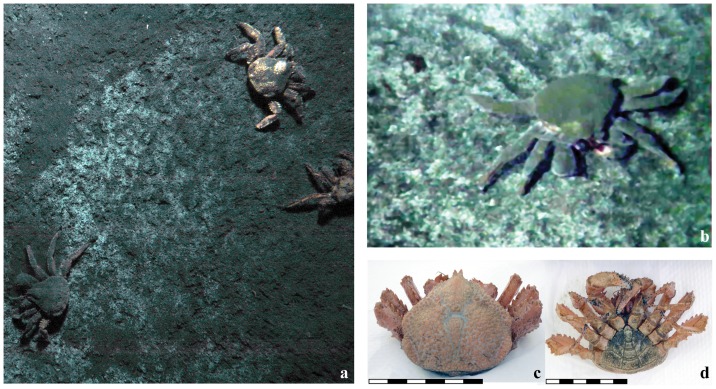
A lithodid crab (*Paralomis diomedeae* relative) commonly encountered at Md. 12. (a) Bird's eye view from a lander mounted still camera (ca. 40×50 cm), (b) close up with visible feeding tracks, (c) dorsal and (d) ventral view of the captured specimen. The scale bars represent 6 cm.

The lithodid crabs were taxonomically identified from photographs that we recorded in situ (i.e., with the deep-sea camera of the DOS lander; e.g. Fig. 1b), and on board from the specimen recovered with Alvin (e.g. Fig. 1c, d). Identification was based on morphological features such as spines, spinules and granules according to our previous work [50].

### Lipid analyses and determination of C and N contents

Extraction of lipids, separation and derivatization was carried out as described previously [51], [52]. Briefly, total lipid extracts (TLEs) were obtained from subsamples of the muscle tissue (∼500 mg wet weight – ww.) and stomach (including its contents; ∼400 mg ww.) by ultrasonication with organic solvent mixtures (methanol and dichloromethane) of decreasing polarity. The TLEs were then saponified and subsequently separated into fractions containing (i) fatty acids (FAs), (ii) hydrocarbons, (iii) ketons and (iv) alcohols (including glycerol ethers). FAs and alcohols were methylated prior to extraction using BF_3_ in methanol and bis(trimethylsilyl)trifluoracetamide (BSTFA) to form fatty acid methyl esters FAMES) and trimethylsilyl (TMS) ethers, respectively. Separation of single lipid compounds, their identification, quantification and the determination of their stable carbon isotope composition was achieved by gas chromatography (GC) coupled to flame ionization detection (GC-FID), quadrupole mass spectrometry with electron ionization (GC-MS) and isotope ratio mass spectrometry (GC-IRMS), respectively [53]. Bulk stable carbon isotope composition was measured from CO_2_, released after flash combustion of ∼100 mg (ww.) of muscle tissue in an automated elemental analyzer (Thermo Flash EA, 1112 Series) coupled to an isotope ratio mass spectrometer (Finnigan Deltaplus XP, Thermo Scientific).

Determination of bulk C and N contents was carried out according to standard methods (www.geomar.de/en/research/fb2/fb2-mg/benthic-biogeochemistry/mg-analytik/determination-of-cns/). Briefly, all inorganic and organic C and N compounds in sediment samples were flash combusted in a CNS analyzer (Carlo Erba Instruments, LTD) and the resulting combustion gases were analyzed with a thermal conductivity detector yielding total C and N contents. Organic C was determined in a similar fashion subsequently to the removal of carbonate-bound C with HCl. C:N-ratios are reported as the molar ratio of organic C versus total N.

### DNA extraction and clone library construction

Total DNA of the microbial community in the crab's stomach was extracted from ∼350 mg (ww.) of stomach material using the FastDNA spin kit for soil (Q-Biogene, USA) as described elsewhere [53]. PCR amplification of 16S rRNA genes, cloning, and sequencing was conducted according to [16]. For the construction of the epsilonproteobacterial clone library, a subsample of 50 µl of formaline-fixed sediment sample (the same sample as used for CARD-FISH, see next section) was centrifuged and the pellet was washed with 1× PBS and finally resuspended in 50 µl H_2_O. Subsequently, we sonicated the sample (2×30 sec, 35 kHz) in a water bath sonicator. 1 µl of a 100-fold dilution was used as template for specific amplification of epsilonproteobacterial 16S rRNA gene sequences using primers Epsi682F (5′ TGTGTAGGGGTAAAATCCG 3′)/GM4. The PCR conditions were as follows: 32 cycles, annealing temperature 44°C. Ten parallel PCRs of each sample were pooled, purified using the QIAquick gel extraction kit (Qiagen, Hilden, Germany) and eluted in 30 µl H_2_O. Cloning reactions were performed with the TOPO TA Cloning Kit (Invitrogen, San Diego, CA, USA) and inserts sequenced using the BigDye Terminator v3.1 Cycle Sequencing Kit (Applied Biosystems, Carlsbad, CA, USA) on an ABI PRISM 3130xl Genetic Analyzer. Sequences were checked for chimeras using the program UCHIME [54] and phylogenetically analyzed with the ARB software package using database SSURef_SILVA_111 (July 2012, 739,633 sequences) downloaded from ARB SILVA resources [55]. The sequence data from the stomach sample will be published in the EMBL, GenBank and DDBJ nucleotide sequence databases under the accession numbers HE974888 to HE974904 as well as HF559372 and HF559373. Sequences from the epsilonproteobacterial clone library will be published under the accession numbers HG321355-HG321366.

### Cell enumeration and catalyzed reporter deposition fluorescence in situ hybridization (CARD–FISH)

Sediment samples for CARD-FISH were fixed in formaldehyde solution, washed in PBS and stored at −20°C as described previously [16]. CARD-FISH was carried out on two parallel surface sediment samples (0–2 cm) from the microbial mat habitat and on one sediment sample from the adjacent, non-covered sediment as described previously [56] with the following modifications: Samples were sonicated before filtration (20 s an amplitude of 42 μm <10 W; MS73 probe, Sonopuls HD70, Bandelin, Germany) and endogenous peroxidases were inactivated by incubation in 0.5% H_2_O_2_ in methanol for 30 min at room temperature. Cell walls were permeabilized with 10 mg ml^−1^ lysozyme in 1×TE-buffer for 45 min at 37°C [57]. For the specific detection of *Epsilonproteobacteria*, we used the HRP-labeled probe Epsi682 (5′-CGGATTTTACCCCTACACM- 3′; biomers.net) [58] applied at 20% formamide. Cells were stained with DAPI, embedded in mounting medium and counted under an epifluorescence microscope in 20–100 independent microscopic fields.

### Methane oxidation- and sulfate reduction rate measurements

Microbial turnover of CH_4_ and SO_4_^2−^ in sediments of Md. 12 was measured with radiotracer assays according to previously published works [51], [59], [60]. Briefly, CH_4_ oxidation and sulfate reduction (SR) rates were determined from 6 push cores distributed over the ∼6 m wide sediment strip covered with bacterial mats and from 3 push cores recovered 1–2 m away from this mat.

## Results and Discussion

### Sea floor observations and biogeochemical environment

#### Sea floor habitat

We visited Md. 12 in 2005 and investigated the seafloor with DSV Alvin and ROV Quest. Visually, we could identify several habitats: reduced sediments covered by whitish microbial mats (e.g. Fig. 1a, b) and adjacent bare sediments without microbial mats (movie S1 in the supplements), colonies of bathymodiolin mussels (*Bathymodiolus* sp.) or siboglinid tubeworms (*Lamellibrachia* sp.) and CH_4_-derived carbonate pavements. As reported previously [47]–[49], these habitats were distributed in a patchy fashion, interspersed by olive-green sediments. The size of the microbial mat patches varied from decimeters to several meters in diameter. The whitish color of the mats suggested that they consisted of thiotrophic bacteria, but the morphology of the mats differed in thickness and structure from those present at most known cold seep systems formed by large sulfur bacteria such as *Beggiatoa, Thiomargarita* or *Thioploca* [6], [15], [53], [61]. They resembled more the *Arcobacter* type mats known from mud volcanoes, such as of the Eastern Mediterranean [14], [62]. The sediments below the mats strongly smelled of sulfide, and previous measurements found ∼15 mM sulfide in porewaters from this habitat [47]. All cores recovered from the microbial mats were also rich in CH_4_ as indicated by their degassing during recovery, and the oversaturated CH_4_ concentrations of ≥1.4 mM in recovered sediments (data not shown). *Ex situ* rate measurements of AOM and SR showed peak values of up to 225 and 327 nmol cm^−3^ d^−1^, and integrated rates of 7.4 and 6.5 mol m^−2^ yr^−1^ for AOM and SR, respectively (Tab. 1). The high sulfide concentrations are thus explained by AOM–dependent SR. We found a high variability in rate measurements when comparing replicates (Tab. 1), possibly related to heterogeneous fluid flow regimes [47]. Just ∼1 m outside the microbial mat habitat, sediments barely smelled of sulfide, and AOM and SR rates were <5 nmol cm^−3^ d^−1^, equivalent to areal rates of <0.3 mol m^−2^ yr^−1^ (Tab. 1).

**Table 1:** Habitat characteristics of Md. 12 sediments covered- and devoid of microbial mats.

	microbial mat	adjacent sediments
tot. No. of crabs observed	184	6
oxidation state	strongly reduced	oxic/anoxic/slightly reduced
organic C (wt%)	2.6 (±0.1)	2.5 (±0.1)
C:N-ratio	9.9 (±0.5)	10.0 (±0.4)
sediment depth of AOM max. (cm)	3 cm	-
AOM max. (nmol cm^−3^ d^−1^)	225 (±60)	5 (±1)
areal AOM (mol m^−2^ yr^−1^)	7.5 (± 1.8)	0.2 (±0.04)
SR max. (nmol cm^−3^ d^−1^)	328 (±107)	8 (±5)
areal SR (mol m^−2^ yr^−1^)	6.5 (±1.8)	0.3 (±0.18)

Total number of crabs was counted from still camera images (2 pictures h^−1^) during an observation period of 408 h. Note that we did not account for feeding tracks without a photo record of the originator and that single specimens could have been counted repeatedly. Org. C contents and C:N-ratios were averaged over the first 10 cm- and AOM and SR rates were integrated over the first 16 cm of surface sediment. Errors are presented as standard error.

#### Lithodid crabs grazing on microbial mats

**Figure 2:**
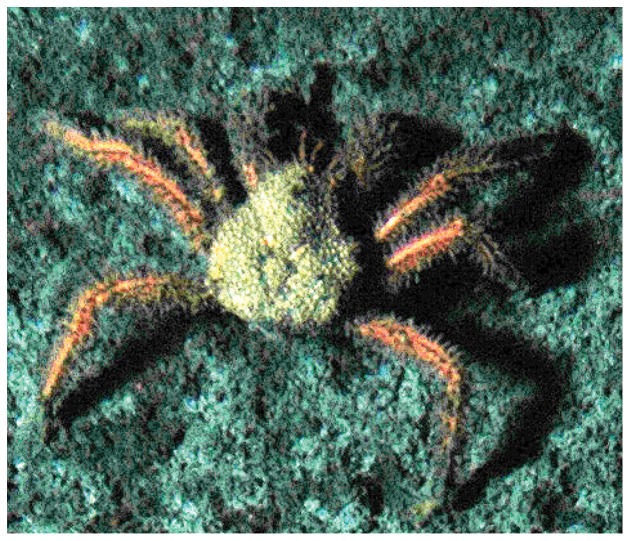
A second type of lithodid crab that we observed rarely at Md. 12 (tentatively identified as *Paralomis papillata* relative).

We frequently observed one type of lithodid crab, which dwelled and apparently fed on the microbial mats of Md. 12 (Fig. 1 a–d, movie S1). Based on the shape of the carapace, rostrum and abdomen documented by high-resolution photography, we identified this species as *Paralomis* sp. [50]. Its morphology is similar to *P. diomedeae*, known to populate continental margins from Costa Rica to Peru, but it differs by the granules on the dorsal carapace surface and the armature of the chelipeds and walking legs. This suggests that the *Paralomis* type of Md. 12 could be a new *Paralomis* species, closely related to *P. diomedeae*. A conclusive determination of the crab's taxonomic status requires collection of new material and in-depth morphological and genetical investigations. We did not conduct off-site surveys during our sampling campaigns so that we can only speculate about the biogeography of the *P. diomedeae* related crabs and potential adaptations for the consumption of chemosynthtic biomass. Little is known about the ecology of *P. diomedeae* but the mouth parts (mandibles, maxillae, maxillulae and maxillipeds) of the previously examined specimens from the eastern Pacific Ocean off Costa Rica [50] and the ones of Md. 12 indicate that both are adapted to an omnivorous diet including detritus. Indeed, during submersible and ROV dives, we observed that the *Paralomis* sp. of Md. 12 grazed on the microbial mats (or on surface sediments including the mats) leaving clearly distinguishable feeding tracks of bare sediments behind (Fig. 1a, b). Other members of the genus *Paralomis*, possibly opportunistic scavengers or predators, have also been observed at other cold seeps and hydrothermal vents constituting a potential link for the export of seep carbon to the surrounding deep sea [28], [63], [64]. However, to our knowledge, only one other publication has reported similar, direct observations from a cold seep setting, i.e. hermit crabs feeding on *Beggiatoa* mats at the Gullfaks seeps, North Sea [65]. The longer-term recordings of the lander-mounted still camera provided further evidence that the microbial mats apparently attracted *Paralomis* sp. (movie S1). During the 408 hours of observation with the lander-mounted camera, we counted 184 sightings of this crab species on a microbial mat patch while only 6 sightings were recorded from surrounding sediments (Tab. 1). Our observations, furthermore, indicate a pattern where intensive grazing was followed by a time period between 8 and 33.5 h of little or no grazing during which the mat regrew until it was grazed of again. We could also record the occurrence of a larger food fall, i.e. a Pyrosome (tunicate colony), which was also consumed by the *Paralomis* sp. (movie S1), confirming that they are opportunistic predators/scavengers. In addition to the *P. diomedeae* relative, we also noticed a second but rather rarely occurring *Paralomis* species (Fig. 2), which we tentatively identified as *P. papillata* or a relative of this species [50]. However, the few available photo documents did not allow for a more reliable identification. One specimen of the so-called Yeti Crab (*Kiwa puravida*) could be seen once on the photo material of the lander mounted camera. We could not observe the *P. papillata* relative or the Yeti Crab feeding on the mats but we noticed snails, which seemed to feed on the mats (movie S1).

### Dietary carbon sources for the Paralomis diomedeae relative

#### Sediment C and N contents

To investigate whether the *P. diomedeae* relative preferentially feeds on microbial mats compared to regular sediments as suggested by our observations, we compared the bulk chemical composition of surface sediments. Both habitat types were characterized by high contents of organic C (∼2.5 weight%) and low C:N-ratios (∼10, Tab. 1) throughout the upper 10 cm of surface sediments. These values are comparable to seafloor sediments from the highly productive upwelling regions of Peru [66] or Chile [67] at ∼1000 m water depth and are indicative for a high fraction of fresh organic matter. This may be explained by the high pelagic primary production in the region of the Costa Rica Dome [68]. The organic deposits in sediments surrounding the seeps of Md. 12 could thus also serve as a relevant carbon source for the *Paralomis sp*. Nevertheless, the nutritional value of the microbial mat is probably higher than that of sediment detritus because of the low C:N-ratio of bacteria (typically 4–5), caused by a relatively high cellular protein content (∼50%) [33]. As the bacterial mat was very thin, the rather coarse sediment sampling of 2 cm sections may thus have masked this signal. Besides the microbial mats and the sedimentary detritus, also the symbiotic megafauna at Md. 12 (i.e., *Bathymodiolus* sp. and *Lamellibrachia* sp.) could be an attractive food source for the crabs. Bivalves and annelids typically contain very high protein contents, which may comprise >70% of their organic matter [34]. However, we did not observe the crabs to feed on these potential food sources.

To further investigate the dietary importance of chemosynthetic vs. photosynthetic carbon for the *Paralomis* sp. we analyzed the molecular signatures of stomach contents, muscle tissue and surface sediments covered by microbial mats (see next 2 sections).

#### 16S rRNA gene libraries and FISH

CARD-FISH analyses of two parallel samples of the microbial mat and underlying sediments with the *Epsilonproteobacteria*-specific probe EPSI682 indicated that *Epsilonproteobacteria* constituted 9.5 and 11.1% of single cells. In contrast, in the surface layer of the adjacent, bare sea floor, we could only detect <2% *Epsilonproteobacteria*. With respect to the morphological appearance of the mat, this confirmed dominance by *Epsilonproteobacteria* rather than by large gammaproteobacterial thiotrophs (*Beggiatoa, Thiomargarita* or *Thioploca*). We used probe EPSI682 as a specific forward primer together with the general bacterial primer GM4 in a PCR to resolve the diversity of CARD-FISH-detected *Epsilonproteobacteria* in the microbial mat habitat. Of the epsilonproteobacterial 16S rRNA genes (53 epsilonproteobacterial sequences from 72 clones analyzed in total, Tab. 2), six sequences grouped within the genus *Sulfurovum*. Other epsilonproteobacterial sequences belonged to the genera *Arcobacter* (25 sequences), *Sulfurimonas* (17 sequences), and *Campylobacter* (5 sequences).

**Table 2:** Epsilonproteobacterial 16S rRNA gene library obtained from surface sediments (0–2 cm) covered with whitish microbial mats.

Order	Family	Genus	No. of clones	Clone representative	Acc. No.
*Campylobacterales*	*Helicobacteraceae*	*Sulfurovum, cluster 1*	6	CRsed_Md12_64_17A3	HG321355
		*Sulfurimonas*	17	CRsed_Md12_64_82B11	HG321360
	*Campylobacteraceae*	*Campylobacter*	5	CRsed_Md12_64_45E6	HG321356
		*Arcobacter*	25	CRsed_Md12_64_66B9	HG321357

From the stomach contents, we could amplify bacterial 16S rRNA gene sequences successfully but repeated attempts to amplify archaeal rRNA genes failed. This likely indicates a very low abundance of archaea in the stomach contents, which is in accordance with our biomarker analyses (see next section). From the amplified bacterial 16S rRNA genes, we analyzed a total of 79 clones. We identified *Epsilonproteobacteria* of the genus *Sulfurovum* as the dominant bacterial group in the stomach of the *Paralomis* sp. (Tab. 3). Two groups (8 and 17 sequences, respectively) with a high intragroup sequence similarity of 98–99% and 94–95% between the two groups were detected. Sequences of cluster 1 were 99.8% similar to sequences from Eel River Basin methane seeps ([69] e.g. acc.no.FJ264599) and those of the second cluster were 97.8% similar to a sequence obtained from particulate detritus from grabs of the vestimentiferan tubeworm *Ridgeia piscesae* (Forget & Jupiter, database release, acc.no. JN662293). Furthermore, sediment *Sulfurovum* sp. was highly similar to the *Sulfurovum* sp. cluster 1 found in the crab's stomach (96.8–99.8% sequence similarity). Also gut and sediment *Campylobacter* spp. showed a high degree of similarity (up to 98.7%). Although *Arcobacter*- and *Sulfurimonas*-related sequences were not retrieved from the crab's stomach, these results provide evidence that *Epsilonproteobacteria* in the stomach originate from the thiotrophic mats, which the crab was observed to feed upon. Together with our observations of crabs feeding specifically on microbial mats, this strongly suggests that these mats are an important nutrition source for the *P. diomedeae* relative recovered from Md. 12.

**Table 3:** Bacterial 16S rRNA gene library obtained from the stomach sample of a lithodid crab (*Paralomis diomedeae* relative), which was observed feeding on surface sediments covered with whitish microbial mats.

Phylum	Class	Order	Family	Genus	# Clones	Clone Repres.	Acc. No.
*Proteobacteria*	*Alphaproteobacteria*	*Rhizobiales*	*Hyphomicrobiaceae*	uncultured	1	ATLA_Crab_Bac_E11	HE974888
	*Gammaproteobacteria*	*Enterobacteriales*	*Enterobacteriaceae*	*Enterobacter*	1	ATLA_Crab_Bac_F06	HE974889
		*Pseudomonadales*	*Moraxellaceae*	*Acinetobacter*	1	ATLA_Crab_Bac_B03	HE974890
	*Deltaproteobacteria*	*Desulfobacterales*	*Desulfobulbaceae*	*Desulfocapsa*	3	ATLA_Crab_Bac_C05	HE974891
				*Desulfobulbus /*Seep-SRB3	2	ATLA_Crab_Bac_H03	HE974892
		*Desulfobacterales*		Seep-SRB2	1	ATLA_Crab_Bac_A02	HF559372
	*Epsilonproteobacteria*	*Campylobacterales*	*Helicobacteraceae*	*Sulfurovum*, cluster 1	8	ATLA_Crab_Bac_H05	HE974893
				*Sulfurovum*, cluster 2	17	ATLA_Crab_Bac_E05	HF559373
			*Campylobacteraceae*	*Campylobacter*	1	ATLA_Crab_Bac_B12	HE974894
*Bacteroidetes*	*Flavobacteria*	*Flavobacteriales*	*Flavobacteriaceae*	*Cloacibacterium*	1	ATLA_Crab_Bac_E03	HE974895
*Planctomycetes*	*Planctomycetacia*	*Planctomycetales*	*Planctomycetaceae*	*Rhodopirellula*	1	ATLA_Crab_Bac_E08	HE974896
*Acidobacteria*	*Acidobacteria*	*Acidobacteriales*	*Acidobacteriaceae*	*uncultured*	1	ATLA_Crab_Bac_A11	HE974897
*Firmicutes*	*Clostridia*	*Clostridiales*	*Lachnospiraceae*	*Cellulosilyticum*	1	ATLA_Crab_Bac_D05	HE974898
	*Bacilli*	*Lactobacillales*			1	ATLA_Crab_Bac_G01	HE974899
*Tenericutes*	*Mollicutes*	*Mycoplasmatales*	*Mycoplasmataceae*	*Lumbricincola and*	25	ATLA_Crab_Bac_D11	HE974900
				*Bacilloplasma relatives*			
*Actinobacteria*	*Actinobacteria*	*Propionibacteriales*	*Propionibacteriaceae*	*Propionibacterium*	2	ATLA_Crab_Bac_C06	HE974901
*Chloroflexi*					1	ATLA_Crab_Bac_G12	HE974902
Candidate Division OD1					6	ATLA_Crab_Bac_A12	HE974903
*Cyanobacteria* (chloroplast)					4	ATLA_Crab_Bac_C07	HE974904

*Epsilonproteobacteria* are known from a variety of hydrothermal vents [70] but have also been found at cold seeps [19], [62], [69], [71] including brines [72], [73]. Members of the genus *Sulfurovum* have been found as free-living bacteria [74], [75], episymbionts associated with a hydrothermal vent shrimp [70], [76] and with the cold seep associated Yeti Crab (*Kiwa puravida*), the latter of which was also found at Md. 12 [26]. Members of the *Sulfurovum* clade were also found in the gut system of the Yeti Crab and a hydrothermal vent shrimp [26], [77]. However, these *Sulfurovum* types shared only ∼95% similarity with our sequences. The biogeochemical functioning of the *Sulfurovum* relatives constituting the microbial mats at Md. 12 is not clear. Known members of the genus *Sulfurovum* use elemental sulfur or thiosulfate as an electron donor, and nitrate or oxygen as electron acceptors [74], [78], [79]. Whole genome sequencing of a *Sulfurovum* strain (NBC37-1) revealed the presence of *sox* genes (coding for enzymes involved in sulfide oxidation) and the strain also had cytoplasmic and periplasmic sulfide-quinone oxidoreductases that oxidize sulfide to elemental sulfur [80].

The stomach contents also contained sequences of other, seep-related chemosynthetic microbes including aerobic organisms thriving in the upmost, oxic surface sediment layer as well as anaerobic strains from deeper sediment layers. We detected one sequence of a relative of *Hypomicrobium* and *Acinetobacter*, which were previously found to grow aerobically on chloro- or dichloromethane [81] and long-chain alkanes [82], respectively. Among the anaerobic strains, we detected two deltaproteobacterial sequences belonging to relatives of the *Desulfobulbus*/Seep-SRB3 cluster, one sequence of the SEEP-SRB2 cluster, which comprise SRB associated to ANME archaea [10], [83], and three sequences related to *Desulfocapsa*, which is a typical SRB in marine sediments, including cold seeps [16]. Furthermore, we also found other bacteria of unknown biogeochemical function that have regularly been found in anoxic cold seep sediments, i.e. relatives of the Candidate Division OD1 and *Propionibacterium* (of which we found six and two sequences, respectively) [11], [84], [85]. However, the relatively low abundance of sequences of anaerobic cold seep microbes indicates that the crab specimen analyzed here mostly fed on oxic surface- and ingested rather little amounts of reduced sediments containing AOM biomass, at least during its last feeding activities. The relatively deep position of the AOM horizon (∼3 cm, Tab. 1) could make archaeal biomass rather inaccessible for the *P. diomedeae* relative or the expectedly high sulfide contents of the AOM horizon [47] could be too toxic.

25 out of 78 sequences were affiliated with *Candidatus Lumbricincola* and *Candidatus Bacilloplasma*, relatives that most likely belong to the gut flora of the *Paralomis* sp. *Candidatus Lumbricincola* has yet only been found in the gut systems of annelids [86]. *Candidatus Bacilloplasma* relatives, on the other hand, were found in the guts of decapod crustaceans (*Scylla* sp.; Sun & Li, database release acc.no. AY360354 and *Nephrops norvegicus* [87]) isopods [88] and chordates (Wu & Wang, database release ac.no. GU293173). Members of the class *Mollicutes* are often pathogenic or parasitic, but also commensal and beneficial associations with their hosts have been found [41], [89].

#### Stable carbon isotope and lipid analyses

**Figure 3:**
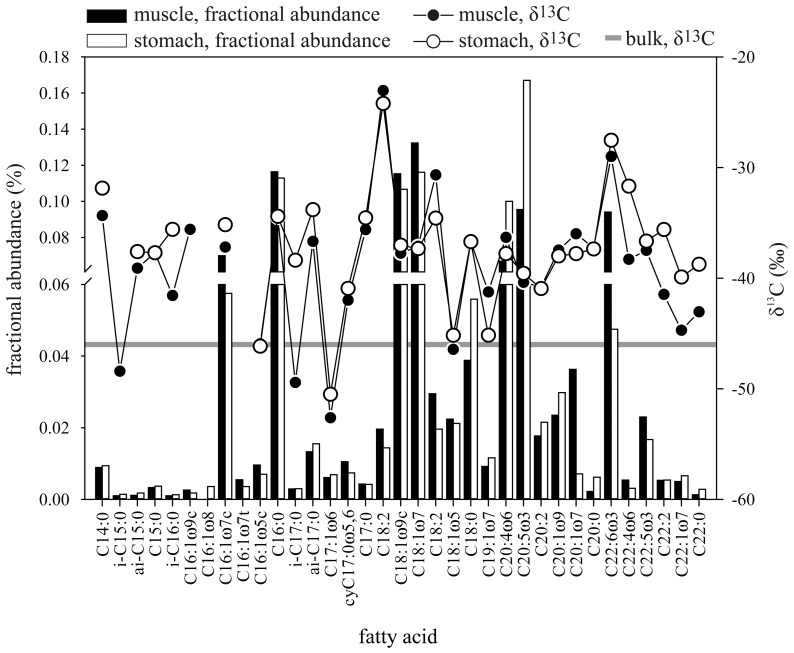
Fractional abundance and stable carbon isotope composition of fatty acids in a muscle- and a stomach sample of the *Paralomis diomedeae* relative. Note that the stomach sample contained stomach contents and stomach epithelium. The bulk stable carbon isotope composition of the muscle is indicated (grey horizontal line).

The bulk stable carbon isotope composition of the muscle tissue was −46‰ (Fig. 3) and thus extremely ^13^C-depleted in comparison to organic matter in regular, recent marine sediments (−10 to −35‰), which are usually of photosynthetic origin (Calvin Benson Cycle) [33]. In eukaryotes, such negative carbon isotope signatures are typically attributed to a methanotrophic food chain [30], [36], [90]–[92]. However, also sulfate reducing bacteria and thiotrophs may show similar signatures by incorporating isotopically depleted CO_2_ derived from methane oxidation and by further fractionation in autotrophic assimilation pathways [93], [94]. Together with our observations of the *Paralomis* sp. feeding habits and the presence of *Sulfurovum* sequences in the crab's stomach, the low δ^13^C-value of the muscle sample thus strongly indicates that the *Paralomis* sp. derives a substantial fraction of organic carbon from the thiotrophic microbial mats, apparently over significant parts of the crab's lifetime. However, the bulk stable isotope composition may also comprise contribution from other chemosynthetic- and/or phototrophic sources.

To investigate the potential dietary carbon sources in more detail, we analyzed lipids from stomach contents (including the stomach epithelium) and from muscle tissue of a walking leg. Only trace amounts of the isoprenoidal glycerol ethers archaeol and *sn2*-hydroxyarchaeol, which are typical for AOM-mediating ANME archaea [95] were found in the stomach sample (data not shown). This directly implies that the stomach of the *Paralomis* sp. contained comparably little archaeal biomass, which is consistent with our 16s rRNA analyses (see above).

Contrary to the archaeal compounds, we detected substantial amounts of FAs in both, the stomach and the muscle sample (Fig. 3, Tab. 4). These lipids are of bacterial and/or eukaryotic origin. In the muscle sample, the FAs may originate from *de novo* synthesis, direct incorporation of food-derived compounds or a mix of both and can thus be used to trace chemosynthetic biomass in heterotrophs [37]. In the stomach sample, these lipids probably originate to a substantial degree from the crabs food source (however, note that the stomach sample contained not only stomach contents but also the stomach epithelium so that it comprises a mixed lipid signature of food and crab). The essential FAs C20∶5ω3, C20∶4ω6 constituted a major fraction of the analyzed FAs, in both samples (Fig. 3). These lipids cannot be synthesized by the crab *de novo* [38] and are thus derived from the crab's food source. With respect to the depleted isotopic signatures of about −40 (C20∶5ω3) and −37‰ (C20∶4ω6), it is very likely that these compounds substantially originate from chemosynthetic bacterial biomass corroborating the molecular, and bulk stable isotope data. Moreover, the higher fractional abundance of C20∶5ω3 and C20∶4ω6 in the stomach- compared to the muscle sample indicates that these FAs were enriched in the stomach contents and thus originate from a recently ingested food source, possibly the microbial mats. Further evidence for the dietary importance of chemosynthetic biomass for the crab is provided by the presence of unusual, ^13^C-depleted FAs in the stomach and the muscle sample (Fig. 3), which contained substantial amounts of the iso- and anteiso-branched C15–C17 FAs, the moneonic FAs C16∶1ω5 and C17∶1ω6 as well as the cyclopropylic FA cyC17∶0ω5,6. Generally, these lipids are not found in crustaceans, but are representative of AOM-associated SRB and/or thiotrophic communities [17], [52], [95], [96]. Just as for the essential FAs C20∶5ω3, C20∶4ω6, the depleted stable carbon isotope signature of these compounds with values as low as −50.5 and −52.6‰ (C17∶1ω6) in the stomach and muscle sample, respectively, point to CH_4_-derived carbon as a dominant carbon source.

**Table 4:** Concentrations (µg g^−1^ dry weight) and stable carbon isotope compositions of fatty acids, cholesterol and desmosterol.

	muscle		stomach	
copound	conc.	δ^13^C	conc.	δ^13^C
∑FA	38.6	−36.3	34.8	−36.5
cholesterol	5.5	−40.8	3.1	−37.5
desmosterol	1.9	−43	0.9	−39.8

The sum of fatty acids comprises all analyzed fatty acids with chain length between 12–22 carbon atoms. The fatty acid stable carbon isotope compositions were calculated as abundance-weighted averages.

In addition to microbial mat biomass, our lipid data provide evidence that the crabs utilize detrital material as well. A second essential FA, C22∶6ω3, had a much higher fractional abundance in the muscle tissue compared to the stomach sample (Fig. 3), which suggests that this compound originates from food sources not present in the stomach at the time of sampling. The high δC^13^C-value of C22∶6ω3 (about −28‰) indicates a photosynthetic origin of this FA. Most likely, the crab had consumed non-seep carbon during past feeding activities, for instance sedimented detrital organic matter, or food falls such as the Pyrosome colony (see movie S1).

In comparison to the bulk stable carbon isotope composition of the muscle tissue (−46‰), the abundance-weighted, average FA δ^13^C-value was considerably less depleted (−36‰, Tab. 4). Therefore, the crab specimen must have consumed additional ^13^C-depleted compounds other than FAs. One such compound class are steroids, of which we found ^13^C-depleted cholesterol (cholest-5-ene-3β-ol) and its probable precursor desmosterol (cholest-5,24-diene-3β-ol) (Tab. 4). Just as the essential FAs, decapod crustaceans appear to lack the ability to synthesize steroids de novo [97], [98] indicating a dietary origin of these compounds. Similar to the essential FA C22∶6ω3, we found a much higher fractional abundance of steroids in the muscle tissue compared to the stomach sample. One source of steroids could be infauna organisms such as polychaetes and nematodes, which, at other cold seeps, were found feeding on organic carbon from deeper sediment layers including the AOM horizon [5]. A second source of steroids could be symbiotic megafauna such as *Bathymodiolus* sp. and *Lamellibrachia* sp, which are also a potential food source for heterotrophic megafauna [36], [99]. We did not measure δ^13^C-values of these organisms at Md. 12, but it is reasonable to assume that the bathymodiolin biomass is strongly ^13^C-depleted just as has been found at other cold seeps [22], [90], [92], so that *Bathymodiolus* sp. could be a source of the crab's ^13^C-depleted steroid pool. *Lamellibrachia* sp., on the other hand, is often not ^13^C-depleted [92], [100]–[102]. Nevertheless, a dietary mixture comprising symbiotic microbial mats, pelagic detritus and megafauna and/or infauna, probably accounts for the difference between bulk- and (abundance weighted) FA stable carbon isotope composition.

## Conclusions

Our sea floor observations together with the analyses of ribosomal RNA genes, lipid biomarkers and stable carbon isotope composition provides evidence that at Md. 12, the lithodid crabs closely related to *Paralomis diomedeae* feed on chemosynthetic biomass. This includes the *Epsilonproteobacteria* (*Sulfurovum* related spp., *Arcobacter* spp. *and Sulfurimonas* spp.), which form the thiotrophic microbial mats at Md. 12. Additionally, our analyses showed that other hydrocarbon degrading- and sulfate-reducing microbes as well as seep macro- and/or megafauna contribute to the nutrition of the crab. The stable carbon isotope- and lipid composition of the crab tissue confirmed that it is an opportunistic scavenger, using both, chemosynthetically as well as photosynthetically derived carbon in its diet. This agrees well with the shape of the crab's feeding appendages, which are functionally similar to other lithodid deep-sea crabs with an omnivorous diet (including detritus) and an opportunistic and vagrant life style. The results of this study suggest that cold seeps may have an important ecological role not only for seep-endemic but also for opportunistic, mobile megafauna.

## Supporting Information

Movie S1 **Time-lapse movie of sea floor observation recorded from a stationary, downward facing camera (2 pictures per hour, field of vision ≈0.4 m^2^).** Lithodid crabs (*Paralomis diomedeae* relative), which were apparently grazing on a thiotrophic, microbial mat were the most common observable fauna type (184 sightings during 408 hours total observation time).
(MP4)

## References

[1] Suess E (1980) Particulate organic carbon flux in the oceans-surface productivity and oxygen utilization. Nature 288: 260–263.

[2] Jahnke RA (1996) The global ocean flux of particulate organic carbon: Areal distribution and magnitude. Global Biogeochem Cy 10: 71–88.

[3] Niemann H, Boetius A (2010) Mud Volcanoes. In: Timmis KN, editors. Handbook of Hydrocarbon and Lipid Microbiology. Berlin: Springer. 205–214.

[4] Suess H (2010) Marine Cold Seeps. In: Timmis KN, editors. Handbook of Hydrocarbon and Lipid Microbiology. Berlin: Springer. 187–204.

[5] Levin LA (2005) Ecology of cold seep sediments: Interactions of fauna with flow, chemistry and microbes. Oceanogr Mar Biol 43: 1–46.

[6] Jørgensen BB, Boetius A (2007) Feast and famine – microbial life in the deep-sea bed. Nat Rev Microbiol 5: 770–781. 17828281 10.1038/nrmicro1745

[7] Dubilier N, Bergin C, Lott C (2008) Symbiotic diversity in marine animals: the art of harnessing chemosynthesis. Nat Rev Microbiol 6: 725–740. 18794911 10.1038/nrmicro1992

[8] Vanreusel A, Andersen AC, Boetius A, Connelly D, Cunha MR, et al (2009) Biodiversity of Cold Seep Ecosystems Along the European Margins. Oceanography 22: 110–127.

[9] Bernardino AF, Levin LA, Thurber AR, Smith C (2012) Comparative Composition, Diversity and Trophic Ecology of Sediment Macrofauna at Vents, Seeps and Organic Falls. PLoS ONE 7: e33515. 22496753 10.1371/journal.pone.0033515 PMC3319539

[10] Knittel K, Boetius A (2009) Anaerobic Oxidation of Methane: Progress with an Unknown Process. Annu Rev Microbiol 63: 311–334. 19575572 10.1146/annurev.micro.61.080706.093130

[11] Holler T, Wegener G, Niemann H, Deusner C, Ferdelman TG, et al (2011) Carbon and sulfur back flux during anaerobic microbial oxidation of methane and coupled sulfate reduction. P Natl Aacad Sci USA 108: E1484–E1490. 10.1073/pnas.1106032108 PMC3248532 22160711

[12] Milucka J, Ferdelman TG, Polerecky L, Franzke D, Wegener G, et al (2012) Zero-valent sulphur is a key intermediate in marine methane oxidation. Nature 491: 541–546. 23135396 10.1038/nature11656

[13] de Beer D, Sauter E, Niemann H, Kaul N, Foucher JP, et al (2006) In situ fluxes and zonation of microbial activity in surface sediments of the Håkon Mosby Mud Volcano. Limnol Oceanogr. 51: 1315–1331.

[14] Omoregie EO, Mastalerz V, de Lange G, Straub KL, Kappler A, et al (2008) Biogeochemistry and community composition of iron- and sulfur-precipitating microbial mats at the Chefren mud volcano (Nile Deep Sea fan, Eastern Mediterranean). Appl Environ Microb 74: 3198–3215. 10.1128/AEM.01751-07 PMC2394935 18378658

[15] Grünke S, Lichtschlag A, de Beer D, Felden J, Salman V, et al (2012) Mats of psychrophilic thiotrophic bacteria associated with cold seeps of the Barents Sea. Biogeosciences 9: 2947–2960.

[16] Knittel K, Boetius A, Lemke A, Eilers H, Lochte K, et al (2003) Activity, distribution, and diversity of sulfate reducers and other bacteria in sediments above gas hydrate (Cascadia margin, Oregon). Geomicrobiol J 20: 269–294.

[17] Niemann H, Lösekann T, de Beer D, Elvert M, Nadalig T, et al (2006) Novel microbial communities of the Haakon Mosby mud volcano and their role as a methane sink. Nature 443: 854–858. 17051217 10.1038/nature05227

[18] Omoregie EO, Niemann H, Mastalerz V, de Lange G, Stadnitskaia A, et al (2009) Microbial methane oxidation and sulfate reduction at cold seeps of the deep Eastern Mediterranean Sea. Mar Geol 261: 114–127.

[19] Niemann H, Fischer D, Graffe D, Knittel K, Montiel A, et al (2009) Biogeochemistry of a low-activity cold seep in the Larsen B area, western Weddell Sea, Antarctica. Biogeosciences 6: 2383–2395.

[20] Childress JJ, Fisher CR, Brooks JM, Kennicutte II MC, Bidigare R, et al (1986) A methanotrophic marine molluscan (Bivalve, Mytilidae) symbiosis: mussels fueled by gas. Science 233: 1306–1308. 17843358 10.1126/science.233.4770.1306

[21] Fisher CR (1990) Chemoautotrophic and Methanotrophic Symbioses in Marine-Invertebrates. Rev Aquat Sci 2: 399–436.

[22] Duperron S, Sibuet M, MacGregor BJ, Kuypers MMM, Fisher CR, et al (2007) Diversity, relative abundance and metabolic potential of bacterial endosymbionts in three Bathymodiolus mussel species from cold seeps in the Gulf of Mexico. Environ Microbiol 9: 1423–1438. 17504480 10.1111/j.1462-2920.2007.01259.x

[23] Petersen JM, Dubilier N (2009) Methanotrophic symbioses in marine invertebrates. Environ Microbiol Rep 1: 319–335. 23765884 10.1111/j.1758-2229.2009.00081.x

[24] Cary SC, Cottrell MT, Stein JL, Camacho F, Desbruyeres D (1997) Molecular identification and localization of filamentous symbiotic bacteria associated with the hydrothermal vent annelid Alvinella pompejana. Appl Environ Microb 63: 1124–1130. 10.1128/aem.63.3.1124-1130.1997 PMC1389137 16535543

[25] Goffredi SK, Waren A, Orphan VJ, Van Dover CL, Vrijenhoek RC (2004) Novel forms of structural integration between microbes and a hydrothermal vent gastropod from the Indian Ocean. Appl Environ Microb 70: 3082–3090. 10.1128/AEM.70.5.3082-3090.2004 PMC404406 15128570

[26] Thurber AR, Jones WJ, Schnabel K (2011) Dancing for Food in the Deep Sea: Bacterial Farming by a New Species of Yeti Crab. PLoS ONE 6: e26243. 22140426 10.1371/journal.pone.0026243 PMC3227565

[27] Tsuchida S, Suzuki Y, Fujiwara Y, Kawato M, Uematsu K, et al (2011) Epibiotic association between filamentous bacteria and the vent-associated galatheid crab, Shinkaia crosnieri (Decapoda: Anomura). J Mar Biol Assoc UK 91: 23–32.

[28] Chevaldonné P, Olu K (1996) Occurrence of anomuran crabs (Crustacea: Decapoda) in hydrothermal vent and cold-seep communities: A review. P Biol Soc Wash 109: 286–298.

[29] Sahling H, Galkin SV, Salyuk A, Greinert J, Foerstel H, et al (2003) Depth-related structure and ecological significance of cold-seep communities - a case study from the Sea of Okhotsk. Deep-Sea Res Pt I 50: 1391–1409.

[30] Sommer S, Linke P, Pfannkuche O, Niemann H, Treude T (2010) Benthic respiration in a seep habitat dominated by dense beds of ampharetid polychaetes at the Hikurangi Margin (New Zealand). Mar Geol 272: 223–232.

[31] Cordes EE, Becker EL, Fisher CR (2010) Temporal shift in nutrient input to cold-seep food webs revealed by stable-isotope signatures of associated communities. Limnol Oceanogr 55: 2537–2548.

[32] Decker C, Olu K (2012) Habitat heterogeneity influences cold-seep macrofaunal communities within and among seeps along the Norwegian margin – Part 2: contribution of chemosynthesis and nutritional patterns. Mar Ecol 33: 231–245.

[33] Madigan MT, Martinko JM, Stahl DA, Clark DP (2012) Brock Biology of Microorganisms. Upper Saddle River: Pearson Prentice Hall. 1152 p.

[34] Canfield DE, Kristensen E, Thamdrup B (2005) Aquatic Geomicrobiology. Oxford: Elsevier. 656 p. 10.1016/S0065-2881(05)48017-7 15797449

[35] Levin LA, Michener RH (2002) Isotopic evidence for chemosynthesis-based nutrition of macrobenthos: The lightness of being at Pacific methane seeps. Limnol Oceanogr 47: 1336–1345.

[36] MacAvoy SE, Carney RS, Fisher CR, Macko SA (2002) Use of chemosynthetic biomass by large, mobile, benthic predators in the Gulf of Mexico. Mar Ecol-Prog Ser 225: 65–78.

[37] MacAvoy SE, Macko SA, Carney RS (2003) Links between chemosynthetic production and mobile predators on the Louisiana continental slope: stable carbon isotopes of specific fatty acids. Chem Geol 201: 229–237.

[38] Bergé J-P, Barnathan G (2005) Fatty Acids from Lipids of Marine Organisms: Molecular Biodiversity, Roles as Biomarkers, Biologically Active Compounds, and Economical Aspects. In: Le Gal Y, Ulber R, editors. Marine Biotechnology I. Springer Berlin/Heidelberg. 49–125. 10.1007/b135782 16566089

[39] Blankenship LE, Yayanos AA (2005) Universal primers and PCR of gut contents to study marine invertebrate diets. Mol Ecol 14: 891–899. 15723681 10.1111/j.1365-294X.2005.02448.x

[40] Meziti A, Kormas K, Pancucci-Papadopoulou M, Thessalou-Legaki M (2007) Bacterial phylotypes associated with the digestive tract of the sea urchin Paracentrotus lividus and the ascidian Microcosmus sp. Russ J Mar Biol 33: 84–91.

[41] Wang W, Gu W, Gasparich GE, Bi KR, Ou JT, et al (2011) Spiroplasma eriocheiris sp. nov., associated with mortality in the Chinese mitten crab, Eriocheir sinensis. Int J Syst Evol Micr 61: 703–708. 10.1099/ijs.0.020529-0 20418415

[42] Mörz T, Fekete N, Kopf A, Brückmann W, Kreiter S, et al. (2005) Styles and productivity of mud diapirism along the middle American margin – Part II: Mound culebra and mounds 11 and 12. In: Martinelli G, Panahi B, editors. Mud Volcanoes, Geodynamics and Seismicity. Dordrecht, the Netherlands: Springer. 49–76.

[43] Kopf A, Deyhle A, Zuleger E (2000) Evidence for deep fluid circulation and gas hydrate dissociation using boron and boron isotopes of pore fluids in forearc sediments from Costa Rica (ODP Leg 170). Mar Geol 167: 1–28.

[44] Ranero CR, von Huene R (2000) Subduction erosion along the Middle America convergent margin. Nature 404: 748–752. 10783885 10.1038/35008046

[45] Sahling H, Masson DG, Ranero CR, Huhnerbach V, Weinrebe W, et al (2008) Fluid seepage at the continental margin offshore Costa Rica and southern Nicaragua. Geochem Geophy Geosy 9: 10.1029/2008GC001978mör.

[46] Hensen C, Wallmann K, Schmidt M, Ranero CR, Suess E (2004) Fluid expulsion related to mud extrusion off Costa Rica – A window to the subducting slab. Geology 32: 201–204.

[47] Linke P, Wallmann K, Suess E, Hensen C, Rehder G (2005) In situ benthic fluxes from an intermittently active mud volcano at the Costa Rica convergent margin. Earth Planet Sc Lett 235: 79–95.

[48] Brückmann W, Bialas J, Kopf A, Rhein M, Rheder G (2009) SUBFLUX, Cruise No. 66, August 12 – December 22, 2005. METEOR-Berichte 09-2. University of Hamburg. 158 p.

[49] Mau S, Sahling H, Rehder G, Suess E, Linke P, et al (2006) Estimates of methane output from mud extrusions at the erosive convergent margin off Costa Rica. Mar Geol 225: 129–144.

[50] Macpherson E, Wehrtmann IS (2010) Occurrence of lithodid crabs (Decapoda, Lithodidae) on the Pacific coast of Costa Rica, Central America. Crustaceana 83: 143–151.

[51] Niemann H, Elvert M, Hovland M, Orcutt B, Judd AG, et al (2005) Methane emission and consumption at a North Sea gas seep (Tommeliten area). Biogeosciences 2: 335–351.

[52] Elvert M, Boetius A, Knittel K, Jørgensen BB (2003) Characterization of specific membrane fatty acids as chemotaxonomic markers for sulfate-reducing bacteria involved in anaerobic oxidation of methane. Geomicrobiol J 20: 403–419.

[53] Niemann H, Duarte J, Hensen C, Omoregie E, Magalhães VH, et al (2006) Microbial methane turnover at mud volcanoes of the Gulf of Cadiz. Geochim Cosmochim Acta 70: 5336–5355.

[54] Edgar RC, Haas BJ, Clemente JC, Quince C, Knight R (2011) UCHIME improves sensitivity and speed of chimera detection. Bioinformatics 27: 2194–2200. 21700674 10.1093/bioinformatics/btr381 PMC3150044

[55] Pruesse E, Peplies J, Glöckner FO (2012) SINA: Accurate high-throughput multiple sequence alignment of ribosomal RNA genes. Bioinformatics 28: 1823–1829. 22556368 10.1093/bioinformatics/bts252 PMC3389763

[56] Pernthaler A, Pernthaler J, Amann R (2002) Fluorescence In Situ Hybridization and Catalyzed Reporter Deposition for the Identification of Marine Bacteria. Appl Environ Microb 68: 3094–3101. 10.1128/AEM.68.6.3094-3101.2002 PMC123953 12039771

[57] Ishii K, Mußmann M, MacGregor BJ, Amann R (2004) An improved fluorescence in situ hybridization protocol for the identification of bacteria and archaea in marine sediments. FEMS Microbiol Ecol 50: 203. 19712361 10.1016/j.femsec.2004.06.015

[58] Moussard H, Corre E, Cambon-Bonavita M-A, Fouquet Y, Jeanthon C (2006) Novel uncultured Epsilonproteobacteria dominate a filamentous sulphur mat from the 13°N hydrothermal vent field, East Pacific Rise. FEMS Microbiol Ecol 58: 449–463. 16989658 10.1111/j.1574-6941.2006.00192.x

[59] Treude T, Boetius A, Knittel K, Wallmann K, Jørgensen BB (2003) Anaerobic oxidation of methane above gas hydrates at Hydrate Ridge, NE Pacific Ocean. Mar Ecol-Prog Ser 264: 1–14.

[60] Jørgensen BB (1978) A comparison of methods for the quantification of bacterial sulfate reduction in coastal marine sediments – I. Measurement with radiotracer techniques. Geomicrobiol J 1: 11–27.

[61] Girnth A-C, Grünke S, Lichtschlag A, Felden J, Knittel K, et al (2011) A novel, mat-forming Thiomargarita population associated with a sulfidic fluid flow from a deep-sea mud volcano. Environ Microbiol. 13: 495–505. 10.1111/j.1462-2920.2010.02353.x 20946529

[62] Grünke S, Felden J, Lichtschlag A, Girnth A-C, De Beer D, et al (2011) Niche differentiation among mat-forming, sulfide-oxidizing bacteria at cold seeps of the Nile Deep Sea Fan (Eastern Mediterranean Sea). Geobiology 9: 330–348. 21535364 10.1111/j.1472-4669.2011.00281.x

[63] Sibuet M, Olu K (1998) Biogeography, biodiversity and fluid dependence of deep-sea cold-seep communities at active and passive margins. Deep-Sea Res Pt II 45: 517–567.

[64] Martin JW, Haney TA (2005) Decapod crustaceans from hydrothermal vents and cold seeps: a review through 2005. Zool J Linn Soc-Lond 145: 445–522.

[65] Hovland M (2007) Discovery of prolific natural methane seeps at Gullfaks, northern North Sea. Geo-Mar Lett 27: 197–201.

[66] Niggemann J, Ferdelman TG, Lomstein BA, Kallmeyer J, Schubert CJ (2007) How depositional conditions control input, composition, and degradation of organic matter in sediments from the Chilean coastal upwelling region. Geochim Cosmochim Acta 71: 1513–1527.

[67] Thamdrup B, Canfield DE (1996) Pathways of carbon oxidation in continental margin sediments off central Chile. Limnol Oceanogr 41: 1629–1650. 11540503 10.4319/lo.1996.41.8.1629

[68] Fiedler PC (2002) The annual cycle and biological effects of the Costa Rica Dome. Deep-Sea Res Pt I 49: 321–338.

[69] Beal EJ, House CH, Orphan VJ (2009) Manganese- and Iron-Dependent Marine Methane Oxidation. Science 325: 184–187. 19589998 10.1126/science.1169984

[70] Campbell BJ, Engel AS, Porter ML, Takai K (2006) The versatile epsilon-proteobacteria: key players in sulphidic habitats. Nat Rev Microbiol 4: 458–468. 16652138 10.1038/nrmicro1414

[71] Omoregie EO, Mastalerz V, de Lange G, Straub KL, Kappler A, et al (2008) Biogeochemistry and community composition of iron- and sulfur-precipitating microbial mats at the Chefren mud volcano (Nile Deep Sea fan, Eastern Mediterranean). Appl Environ Microb 74: 3198–3215. 10.1128/AEM.01751-07 PMC2394935 18378658

[72] Joye SB, Samarkin VA, Orcutt BN, MacDonald IR, Hinrichs KU, et al (2009) Metabolic variability in seafloor brines revealed by carbon and sulphur dynamics. Nat Geosci 2: 349–354.

[73] Borin S, Brusetti L, Mapelli F, D'Auria G, Brusa T, et al (2009) Sulfur cycling and methanogenesis primarily drive microbial colonization of the highly sulfidic Urania deep hypersaline basin. P Natl Aacad Sci USA 106: 9151–9156. 10.1073/pnas.0811984106 PMC2685740 19470485

[74] Inagaki F, Takai K, Nealson KH, Horikoshi K (2004) Sulfurovum lithotrophicum gen. nov., sp. nov., a novel sulfur-oxidizing chemolithoautotroph within the ε-Proteobacteria isolated from Okinawa Trough hydrothermal sediments. Int J Syst Evol Micr 54: 1477–1482. 10.1099/ijs.0.03042-0 15388698

[75] Schauer R, Roy H, Augustin N, Gennerich HH, Peters M, et al (2011) Bacterial sulfur cycling shapes microbial communities in surface sediments of an ultramafic hydrothermal vent field. Environ Microbiol 13: 2633–2648. 21895907 10.1111/j.1462-2920.2011.02530.x

[76] Tokuda G, Yamada A, Nakano K, Arita NO, Yamasaki H (2008) Colonization of Sulfurovum sp. on the gill surfaces of Alvinocaris longirostris, a deep-sea hydrothermal vent shrimp. Mar Ecol 29: 106–114.

[77] Durand L, Zbinden M, Cueff-Gauchard V, Duperron S, Roussel EG, et al (2010) Microbial diversity associated with the hydrothermal shrimp Rimicaris exoculata gut and occurrence of a resident microbial community. FEMS Microbiol Ecol 71: 291–303. 19951370 10.1111/j.1574-6941.2009.00806.x

[78] Takai K, Campbell BJ, Cary SC, Suzuki M, Oida H, et al (2005) Enzymatic and genetic characterization of carbon and energy metabolisms by deep-sea hydrothermal chemolithoautotrophic isolates of Epsilonproteobacteria. Appl Environ Microb 71: 7310–7320. 10.1128/AEM.71.11.7310-7320.2005 PMC1287660 16269773

[79] Yamamoto M, Nakagawa S, Shimamura S, Takai K, Horikoshi K (2010) Molecular characterization of inorganic sulfur-compound metabolism in the deep-sea epsilonproteobacterium Sulfurovum sp. NBC37-1. Environ Microbiol 12: 1144–1153. 20132283 10.1111/j.1462-2920.2010.02155.x

[80] Nakagawa S, Takaki Y, Shimamura S, Reysenbach AL, Takai K, et al (2007) Deep-sea vent epsilon-proteobacterial genomes provide insights into emergence of pathogens. P. Natl Aacad Sci USA 104: 12146–12150. 10.1073/pnas.0700687104 PMC1907315 17615243

[81] Fetzner S (2010) Aerobic Degradation of Halogenated Aliphatics. In: Timmis KN, editors. Handbook of Hydrocarbon and Lipid Microbiology. Springer. 866–885.

[82] Rojo S (2010) Enzymes for Aerobic Degradation of Alkanes. In: Timmis KN, editors. Handbook of Hydrocarbon and Lipid Microbiology. Springer. 782–797.

[83] Kleindienst S, Ramette A, Amann R, Knittel K (2012) Distribution and in situ abundance of sulfate-reducing bacteria in diverse marine hydrocarbon seep sediments. Environ Microbiol 14: 2689–2710. 22882476 10.1111/j.1462-2920.2012.02832.x

[84] Lanoil BD, Sassen R, La Duc MT, Sweet ST, Nealson KN (2001) *Bacteria* and *Archaea* physically associated with Gulf of Mexico gas hydrates. Appl Environ Microb 67: 5143–5153. 10.1128/AEM.67.11.5143-5153.2001 PMC93283 11679338

[85] Bowles MW, Samarkin VA, Bowles KM, Joye SB (2011) Weak coupling between sulfate reduction and the anaerobic oxidation of methane in methane-rich seafloor sediments during ex situ incubation. Geochim Cosmochim Acta 75: 500–519.

[86] Nechitaylo TY, Timmis KN, Golyshin PN (2009) ‘Candidatus Lumbricincola’, a novel lineage of uncultured Mollicutes from earthworms of family Lumbricidae. Environ Microbiol 11: 1016–1026. 19396950 10.1111/j.1462-2920.2008.01837.x

[87] Meziti A, Ramette A, Mente E, Kormas KA (2010) Temporal shifts of the Norway lobster (Nephrops norvegicus) gut bacterial communities. FEMS Microbiol Ecol 74: 472–484. 20831590 10.1111/j.1574-6941.2010.00964.x

[88] Kostanjšek R, Strus J, Avgustin G (2007) “andidatus Bacilloplasma,”a novel lineage of Mollicutes associated with the hindgut wall of the terrestrial isopod Porcellio scaber (Crustacea : Isopoda). Appl Environ Microb 73: 5566–5573. 10.1128/AEM.02468-06 PMC2042062 17630315

[89] Whitcomb RF (1981) The Biology of Spiroplasmas. Annu Rev Entomol 26: 397–425.

[90] Paull CK, Jull AJT, Toolin LJ, Linick T (1985) Stable isotope evidence for chemosynthesis in an abyssal seep community. Nature 317: 709–711.

[91] Van Dover CL (2007) Stable Isotope Studies in Marine Chemoautotrophically Based Ecosystems: An Update. In: Michener R, Lajtha K, editors. Stable Isotopes in Ecology and Environmental Science. Blackwell Publishing Ltd. 202–237.

[92] Thurber AR, Kröger K, Neira C, Wiklund H, Levin LA (2010) Stable isotope signatures and methane use by New Zealand cold seep benthos. Mar Geol 272: 260–269.

[93] Boetius A, Ravenschlag K, Schubert C, Rickert D, Widdel F (2000) A marine microbial consortium apparently mediating anaerobic oxidation of methane. Nature 407: 623. 11034209 10.1038/35036572

[94] Lösekann T, Robador A, Niemann H, Knittel K, Boetius A, et al (2008) Endosymbioses between bacteria and deep-sea siboglinid tubeworms from an Arctic Cold Seep (Haakon Mosby Mud Volcano, Barents Sea). Environ Microbiol 10: 3237–3254. 18707616 10.1111/j.1462-2920.2008.01712.x

[95] Niemann H, Elvert M (2008) Diagnostic lipid biomarker and stable carbon isotope signatures of microbial communities mediating the anaerobic oxidation of methane with sulphate. Org Geochem 39: 1668–1677.

[96] Bergé J-P, Barnathan G (2005) Fatty Acids from Lipids of Marine Organisms: Molecular Biodiversity, Roles as Biomarkers, Biologically Active Compounds, and Economical Aspects. In: Le Gal Y, Ulber R, editors. Marine Biotechnology I. Springer Berlin/Heidelberg. 49–125. 10.1007/b135782 16566089

[97] Blumenberg M, Seifert R, Reitner J, Pape T, Michaelis W (2004) Membrane lipid patterns typify distinct anaerobic methanotrophic consortia. P Natl Aacad Sci USA 101: 11111–11116. 10.1073/pnas.0401188101 PMC503748 15258285

[98] Van den Oord A (1964) The absence of cholesterol synthesis in the crab, *Cancer Pagurus* L. Comp Biochem Physiol. 13: 461–467. 10.1016/0010-406x(64)90038-6 14246359

[99] MacAvoy SE, Fisher CR, Carney RS, Macko SA (2005) Nutritional associations among fauna at hydrocarbon seep communities in the Gulf of Mexico. Mar Ecol-Prog Ser 292: 51–60.

[100] Kennicutt MC, Brooks JM, Bidigare RR, Fay RR, Wade TL, et al (1985) Vent-type taxa in a hydrocarbon seep region on the Louisiana slope. Nature 317: 351–353.

[101] MacAvoy SE, Macko SA, Joye SB (2002) Fatty acid carbon isotope signatures in chemosynthetic mussels and tube worms from Gulf of Mexico hydrocarbon seep communities. Chem Geol 185: 1–8.

[102] MacDonald IR, Boland GS, Baker JS, Brooks JM, Kennicutt MC, et al (1989) Gulf of Mexico hydrocarbon seep communities. Mar Biol 101: 235–247.

